# Presence of *egc*-positive major clones ST 45, 30 and 22 among methicillin-resistant and methicillin-susceptible oral *Staphylococcusaureus* strains

**DOI:** 10.1038/s41598-020-76009-1

**Published:** 2020-11-03

**Authors:** Ewa Kwapisz, Katarzyna Garbacz, Maja Kosecka-Strojek, Justyna Schubert, Jacek Bania, Jacek Międzobrodzki

**Affiliations:** 1 Department of Oral Microbiology, Medical Faculty, Medical University of Gdansk, Gdansk, Poland; 2 Department of Microbiology, Faculty of Biochemistry, Biophysics and Biotechnology, Jagiellonian University, Krakow, Poland; 3 Department of Food Hygiene and Consumer Health Protection, Wroclaw University of Environmental and Life Sciences, Wroclaw, Poland

**Keywords:** Clinical microbiology, Bacterial infection

## Abstract

The oral cavity may comprise a significant reservoir for *Staphylococcus aureus* but the data on molecular epidemiology and clonal distribution of oral strains are really scarce. This study aimed to evaluate the clonal relatedness in *S. aureus* isolated from oral cavity and their relationship with carriage of virulence genes, and antimicrobial resistance profiles. A total of 139 oral *S. aureus* isolates were obtained from 2327 analysed oral samples of dental patients. Antimicrobial susceptibility testing was performed. Isolates were characterized using protein A gene (*spa*) typing, *spa*-CC clonal complexes, toxin genes and SCC*mec* typing for MRSA. High resistance rates for penicillin, tetracycline and gentamicin were detected, respectively 58.3%, 42.4%, and 35.2%. Twelve (8.6%) *S. aureus* isolates were identified as MRSA. All of MRSA isolates were *mec*A-positive and *mec*C-negative. SCC*mec* IV was the most common type (66.7%), which was typical for community-acquired MRSA (CA-MRSA). Overall, the enterotoxin gene cluster (*egc*) was the most frequent detected virulence factor (44.9%), both in MSSA and MRSA isolates. Presence of genes encoding for the enterotoxins (*sea*, *seb*, *sec*, *seh*, *sek*), exfoliative toxin A (*eta*), and toxic shock syndrome toxin-1 (*tst*) was also observed. Strains carrying *lukS-*PV*/lukF-*PV genes belonged to SCC*mec*V- *spa* type t437. The most prevalent *spa* types were t091, t015, t084, t002, t571, and t026 among all 57 identified. *Spa* types, including 3 new ones, grouped in 6 different *spa*-CC clonal complexes, with four major dominated; CC45, CC30, CC5, and CC15. This study demonstrated that both methicillin-susceptible and methicillin-resistant major European clones of *S. aureus* could be isolated from the oral cavity of dental patients, with the emergence of PVL-positive CA-MRSA strains. The oral cavity should be considered as a possible source of toxigenic *egc-*positive *S. aureus* strains, in terms of potential risk of cross-infection and dissemination to other body sites.

## Introduction

*Staphylococcus aureus* is responsible for a wide variety of human infections ranging from mild symptoms in superficial skin infections to life-threatening systemic disease, such as infective endocarditis and sepsis^1^.

*Staphylococcus aureus* may also be associated with some oral conditions and infections in dentistry. These include angular cheilitis, mucositis, some endodontic infections, osteomyelitis of the jaw, and parotitis^2–4^. Recent reports suggest that *S. aureus* is possibly also involved in the pathogenesis of periodontal lesions^5,6^.

However, the role of *S. aureus* in oral infections still raises some controversies since the presence of *S. aureus* in the oral cavity has the commensal asymptomatic character in the healthy carriers^7,8^. Although anterior nares are considered a primary ecological niche for *S. aureus,* it is estimated that 15–50% of persons colonized by these microorganisms are non-nasal carriers^9^. The oral cavity is frequently colonized by *S. aureus,* either as a primary location or as a consequence of migration from the anterior nares^10,11^. Recent evidence suggests that the colonization may be not only transient but also persistent^12,13^. According to Kearney, the oral cavity represents a significant and underappreciated reservoir for *S. aureus*^10^.

The oral carriage may give rise to staphylococcal infection, whether endogenous or cross-infection. Oral carriers, especially immunocompromised persons, such as hemodialyzed patients, subjects with haematological malignancies, rheumatoid arthritis, diabetes mellitus, etc., have increased risk of severe endogenous infections^14–17^. According to Terpenning et al., the presence of *S. aureus* in saliva was a significant risk factor for aspiration pneumonia^18^. Also, a strong relationship was found between the isolation of *S. aureus* and the occurrence of other severe infections, such as bacteraemia and infective endocarditis^19–21^. As the majority of these infections are endogenous, the risk among *S. aureus* colonized patients is 11.5 times higher than in non-colonized persons^22^.

Furthermore, the oral cavity may also constitute a reservoir for transmission events^23^. Small et al. emphasized that this site can be often overlooked for screening and subsequent decolonization^24^. A large body of evidence suggests that the transmission of *S. aureus* may occur between patients and dentist via the clinical environment^3,4,25^.

Thus understanding the distribution and relatedness of staphylococcal clones colonizing oral cavity is essential for the strategies to control its dissemination and to reduce the incidence of infections^26^.

Currently, genotyping using protein A gene (*spa*) typing is the most popular method for the epidemiological analysis of *S. aureus* isolates, their genetic relatedness and diversity^27,28^. *spa* typing is a useful tool for discriminating *S. aureus* from different sources and nosocomial infection control^29^. The method is based on *spa* gene polymorphism in the X-region, with variable number of 24-bp repeat sequences. The results of *spa* typing correlate with other genotyping methods, especially with worldwide used clonal grouping based on multilocus sequence typing (MLST)^30,31^.

The studies of molecular epidemiology and clonal distribution of *S. aureus* isolated from the oral cavity are really scarce. This study aimed to evaluate the clonal relatedness in *S. aureus* isolated from oral cavity and their relationship with carriage of virulence genes, as well as antimicrobial resistance profiles.

## Results

### Prevalence of *S. aureus* isolates

A total 139 *S. aureus* were isolated from 2327 oral samples of 750 (18.5%) dental patients with symptoms of infection. *S. aureus* were detected among 128 patients, 71 females and 57 males aged between 17 and 82 years (mean 56 years). The number of collected samples from each patients varied, from one sample per patients (118 patients) to two (9 patients) or three samples (1 patient). Sixty seven isolates derived from dorsum of the tongue swabs, 38 isolates from buccal mucosa swabs, 19 isolates from denture swabs and 15 isolates from the corners of the mouth swabs.

### Antimicrobial susceptibility

Overall, 139 isolates demonstrated resistance to penicillin (58.3%), tetracycline (42.4%), gentamicin (35.2%), clindamycin (19.4%), erythromycin (19.4%), amoxicillin/clavulanic acid (16.8%), cefoxitin (8.6%), oxacillin (8.6%), chloramphenicol (4.3%), trimethoprim/sulfamethoxazole (4.3%), and ciprofloxacin (1.4%). None of the isolates were resistant to vancomycin. A high proportion (68%) of resistance to gentamicin obtained in disk diffusion method was verified by E-tests (35.2%). D-test demonstrated that 15.1% of isolates represented the inducible phenotype of clindamycin resistance (MLSB_i_) (Table 1).

**Table 1:** Antibiotic resistance of MRSA, MSSA and the 6 *spa*-clonal complexes (*spa*-CC) of oral *S. aureus* isolates.

Antibiotic	MSSA % n = 127	MRSA% n = 12	spa-CC 015 n = 14	spa-CC 021 n = 11	spa-CC 571/1451 n = 10	spa-CC 084 n = 10	spa-CC 888 n = 5	spa-CC267 n = 4	p-value MRSA vs. MSSA	p-value spa-CC groups
Cefoxitin	0	100 (12)	0	9.1 (1)	0	10 (1)	40 (2)	0	< 0.001	0.072
Penicillin	54.3 (69)	100 (12)	21.4 (3)	100 (11)	40 (4)	90 (9)	60 (3)	50 (2)	0.001	0.001
Amoxicillin/clavulanic acid	8.7 (11)	100 (12)	0	0	10 (1)	0	0	50( 2)	< 0.001	0.003
Erythromycin	18.9 (24)	25 (3)	0	54.5 (6)	50 (5)	10 (1)	40 (2)	0	0.423	0.007
Clindamycin	4.7 (6)	0	7.1 (1)	0	10 (1)	0		0	0.441	0.742
Clindamycin_ind_^a^	14.2 (18)	25 (3)	0	54.5 (6)	50 (5)	10 (1)	0	0	0.391	0.002
Ciprofloxacin	1.6 (2)	0	0	0	0	0	0	0	0.661	–
Gentamicin	37 (47)	16.6 (2)	50 (7)	36.4 (4)	20 (2)	10 (1)		0	0.214	0.091
Tetracycline	42.5 (54)	41.7 (5)	7.1 (1)	81.8 (9)	30 (3)	20 (2)	40 (2)	100 (4)	0.954	< 0.001
Chloramphenicol	4.7 (6)	0	0	27.3 (3)	0	0	0	0	0.441	0.029
Trimethoprim/sulfamethoxazole	3.9 (5)	8.3 (1)	0	0	0	0	0	0	0.424	–
Multidrug resistance	26 (33)	41.7 (5)	0	63.6 (7)	40% (4)	10 (1)	0	0	0.309	0.001

^a^Clindamycin_ind_ inducible clindamycin resistance, MRSA methicillin-resistant *S. aureus*; MSSA methicillin-sensitive *S. aureus.*

Twelve (8.6%) *S. aureus* isolates were identified as MRSA. All of MRSA isolates were *mec*A-positive, while none harboured *mec*C. Twenty-five percent of isolates showed MLSB_i_ resistance and this proportion was higher than in MSSA isolates. Resistance to tetracycline, clindamycin, erythromycin, gentamicin, and trimethoprim/sulfamethoxazole was found in 41.7%, 25%, 25%, 16.6% and 8.3% of the MRSA isolates, respectively. All isolates were sensitive to ciprofloxacin, chloramphenicol, and vancomycin (Table 1). Staphylococcal cassette chromosome *mec* (SCC*mec*) types IV (66.7%) and V (33.3%) were detected, suggesting a community origin (CA-MRSA). No isolate represented I, II and III SCC*mec* types.

Multidrug-resistance (MDR) was observed among 38 (27.3%) of all isolated *S. aureus*, in MRSA turned out to be higher (41.7%) than in MSSA (26%). MDR isolates were resistant to 3, 4 and 5 groups of antibiotics; 57.9%, 18.4%, and 23.7% respectively.

### Distribution of toxin genes

Analysis of the distribution of virulence genes among the 139 oral *S. aureus* isolates evidenced that 66.9% of them contained genes encoding toxins, with a high percentage in MRSA isolates (83.3%). Overall, the gene cluster *egc* (*seg, sei, sem, sen, seo, seu*) were the most common detected superantigen (44.9%). Genes encoding for the enterotoxins *sea* (9.4%), *seb* (3.6%), *sec* (12.9%), *seh* (2.2%), *sek* (3.6%), exfoliative toxin A *eta* (2.2%), and toxic shock syndrome toxin-1 *tst* (7.2%) were also identified. None of the isolates tested positively for *sed* and *see* enterotoxin genes. Detection of the Panton-Valentine leukocidin genes (*lukS*-PV/*lukF*-PV) (p = 0.007), the enterotoxin genes *seb* and *sek* were significantly more prevalent (16.6%) among MRSA than among MSSA isolates. While enterotoxin genes *sea, seh, sej*, *sel* and exfoliative toxin B gene (*etb*) were solely present in MSSA isolates, 10.2%, 2.4%, 9.4%, 4.7%, 0.8%, respectively (Table 2).

**Table 2:** Prevalence of toxin genes among oral *S. aureus* isolates.

Toxin genes^a^	MSSA % (n = 127)	MRSA % (n = 12)	Total % (n = 139)	p value
*sea*	10.2 (13)	0	9.4 (13)	0.601
*seb*	2.4 (3)	16.6 (2)	3.6 (5)	0.059
*sec*	11.8 (15)	25 (3)	12.9 (18)	0.166
*sed*	0	0	0	–
*see*	0	0	0	–
*seg*	44.9 (57)	41.6 (5)	44.6 (62)	0.830
*seh*	2.4 (3)	0	2.2 (3)	0.590
*sei*	44.9 (57)	41.6 (5)	44.6 (62)	0.830
*sej*	9.4 (12)	0	8.6 (12)	0.599
*sek*	2.4 (3)	16.6 (2)	3.6 (5)	0.059
*sel*	4.7 (6)	0	3.6 (6)	0.441
*sem*	42.5 (54)	33.3 (4)	41.7 (58)	0.761
*sen*	42.5 (54)	33.3 (4)	40.3 (56)	0.762
*seo*	44.1 (56)	33.3 (4)	43.2 (60)	0.553
*seu*	40.1 (51)	16.6 (2)	38.1 (53)	0.131
*eta*	1.6 (2)	8.3 (1)	2.2 (3)	0.239
*etb*	0.8 (1)	0	0.7 (1)	0.758
*lukS*-PV/*lukF*-PV	0	16.6 (2)	1.4 (2)	0.007
*tst*	7 (9)	8.3 (1)	7.2 (10)	0.873
Total	65.4 (83)	83.3 (10)	66.9 (93)	0.337

^a^Genes encoding staphylococcal enterotoxins (*sea, seb, sec, sed, see, seg, she, sei, sej, sek, sel, sem, sen, seo, seu*), exfoliative toxins (*eta,etb*), Panton-Valentine leukocidin (*lukS*-PV/*lukF*-PV), and toxic shock syndrome toxin-1 (*tst*).

### spa typing

The *spa* typing analysis revealed distinct 57 *spa* types within 139 tested *S. aureus* isolates, the most common were: t091 (10.8%), t015 (7.9%), t002 (5.0%), t012 (5.0%), t084 (4.3%), t005 (3.6%), t230 (2.9%), t571 (2.2%) ,t026 (2.2%). t065 (2.2%), t148 (2.2%), t160 (2.2%), and t242 (2.2%). The other *spa* types were less frequent, such as t045, t056, t085, t127, t131, t209, t437, t688, t693, t711, t1268, and t3297. The determined *spa* types included three new ones: t18952, t18953, and t18954; all of them were registered in the international database, Ridom SpaServer (https://spaserver.ridom.de/) (Table 3).

**Table 3:** *spa* types, clonal complexes, genetic profile and antimicrobial resistance of oral *S. aureus* isolates.

No of cluster	*spa*-CC	MLST-CC	Predicted ST	*spa* type	Toxin genes	Antimicrobial resistance	n
1	*spa*-CC 571/1451	CC 45	ST-45	t230	*egc* (4)	P,CC,TE (1); P (2);	4
1	*spa*-CC 571/1451			t571	*seh* (1)	E,CCind (2); E,CCind,GM (1)	3
1	*spa*-CC 571/1451			t1451	None	E,CCind,GM,TE (1)	1
1	*spa*-CC 571/1451			t3307	None	E,CCind,TE (1)	1
1	*spa*-CC 571/1451			t5529	*sec,egc* (1)	P,AMC (1)	1
2	*spa*-CC 021	CC30	ST 30	t012	*egc,tst* (1); *sea,egc* (6)	SCC*mec* V-FOX,P,AMC,TE, (1); P,E,CCind,TE,C (3); P,E,CCind,GM,TE (3);	7
2	*spa*-CC 021	CC30	ST 30	t021	*sea*, *tst* (1)	P (1)	1
2	*spa*-CC 021	CC30	ST 30	t318	*egc* (1)	P,TE (1)	1
2	*spa*-CC 021			t342	*sea* (1)	P,GM,TE (1)	1
2	*spa*-CC 021			t2141	*tst* (1)	P (1)	1
3	*spa-*CC084	CC15	ST15	t084	*egc* (1)	P,E,CCind,TE (1); P (4); E (1);	6
3	*spa-*CC084	CC15	ST15	t085	None	P,TE (1); P,GM (1)	2
3	*spa-*CC084			t120	None	P (1)	1
3	*spa-*CC084			t13670	None	SCC*mec* IV-FOX,P,AMC, (1)	1
4	*spa-*CC888		ST12	t156	*sec* (1)	SCC*mec* IV-FOX,P,AMC, (1)	1
4	*spa-*CC888		ST12	t160	*seb,sek* (2)	E,TE (2); P (1)	3
4	*spa-*CC888			t888	*sec* (1)	SCC*mec* V-FOX,P,AMC, (1)	1
5	*spa-*CC015	CC45	ST45	t015	*sec,egc,sej,sel* (4); *sec,egc,sel* (1); *egc,sel* (1); *sec* (2) ;*egc* (3);	GM,TE (1); GM (6);	11
5	*spa-*CC015			t630	*sec* (1)	P,CC (1)	1
5	*spa-*CC015			t1268	*sec,egc* (2)	P (2)	2
6	*spa-*CC267			t267	None	P,AMC,TE (1)	1
6	*spa-*CC267			t359	None	P,AMC,TE (1)	1
6	*spa-*CC267			t3297	None	TE (2)	2
7	No founder		ST121	t159	*egc,eta,etb* (1)	P (1)	1
7	No founder			t645	*egc* (1)	P,AMC,E,TE (1)	1
8	No founder	CC22	ST22	t005	*egc* (4)	P (1); GM (2)	5
8	No founder			t790	*egc* (1)	P,TE (1)	1
9	No founder	CC45	ST45	t065	*egc* (3)	SXT (3)	3
9	No founder			t560	*egc* (1)	P (1)	1
10	No founder	CC5	ST5	t002	*egc* (2); *egc,sej* (2)	P,AMC (1); GM,TE,C (1); P,GM,TE (2); GM,TE (3)	7
10	No founder			t242	*egc,tst* (3)	P,TE (3)	3
11	No founder	CC8	ST8	t008	*sea,sej* (1)	P (1)	1
11	No founder			t1187	None	P,TE (1)	1
Singleton	Singleton	CC5	ST5	t045	None	E,CCind,GM,TE (2)	2
Singleton	Singleton		ST101	t056	None	GM,TE (2)	2
Singleton	Singleton		ST7	t091	*egc* (3); *sej* (4)	SCC*mec* IV-FOX,P,AMC (1); P,AMC,GM (1) P,GM,TE (3); P,GM (1); P,CC (1); CC,TE (1); P(1); CC (2); GM (3)	15
Singleton	Singleton	CC1	ST1	t127	*seh* (2)	P,GM,TE (1); P,TE (1)	2
Singleton	Singleton			t131	None	TE (2)	2
Singleton	Singleton			t148	*egc* (3)	P,AMC,CC,GM (1); P,AMC (2)	3
Singleton	Singleton			t189	*egc*	SCC*mec*IV-FOX,P,AMC,GM,TE (1)	1
Singleton	Singleton		ST109	t209	*egc,eta* (1); *sea* (1)	P,E,CCind,TE(1); P,GM (1)	2
Singleton	Singleton			t254	None	P,TE (1)	1
Singleton	Singleton			t437	*seb,sek*,*luk*S-PV/*luk*F-PV(2)	SCC*mec* V-FOX,P,AMC,E,CCind (1); SCC*mec* V-FOX,P,AMC,TE,SXT (1)	2
Singleton	Singleton			t688	*egc* (1)	E,C,TE (2)	2
Singleton	Singleton			t711	*sea* (1)	GM (1)	2
Singleton	Singleton			t1773	None		1
Singleton	Singleton			t4132	None	P,CIP,TE,GM,STX (2)	2
Singleton	Singleton			t5644	*sec* (2)	SCC*mec* IV-FOX,P,AMC,GM (1)	2
Singleton	Singleton			t18117	*seb,sek* (1)	TE (1)	1
Singleton	Singleton			18952	*tst* (1)	P,CC,GM (1)	1
Singleton	Singleton			18953	*egc* (2)	SCC*mec* IV-FOX,P,AMC (1); P,GM (1);	2
Singleton	Singleton			18954	*sea,egc,tst* (1)	P,TE (1)	1
Excluded	Excluded	CC45	ST45	t026	*sec,egc* (1); *sea,tst* (1)	P (1); GM (1)	3
Excluded	Excluded			t282	*egc* (2)	P,AMC,E,CCind (2)	2
Excluded	Excluded			t693	*egc,eta* (1); *egc* (1)	SCC*mec* IV-FOX,P,AMC,E,CCind,TE, (2)	2
Excluded	Excluded			t1509	*sec* (1)		1
Excluded	Excluded			t17293	*sec* (2)		2
NT	NT			NT	*egc,sej* (1); *egc*(1)*; tst* (1)	P,E,CCind,GM,TE (1); P,GM,TE (1); P,GM (2), P,E (1)	5

*spa-CC* *spa* clonal complex, *MLST* multi-locus sequence typing, *NT* non-typeable, *FOX* cefoxitin, *P* penicillin, *AMC* amoxicillin/clavulanic acid, *E* erythromycin, *CC* clindamycin, *CCind* clindamycin inducible resistance (iMLS_B_), *CIP* ciprofloxacin, *GM* gentamicin, *TE* tetracycline, *C* chloramphenicol, *SXT* trimethoprim–sulfamethoxazole.

**Figure 1:**
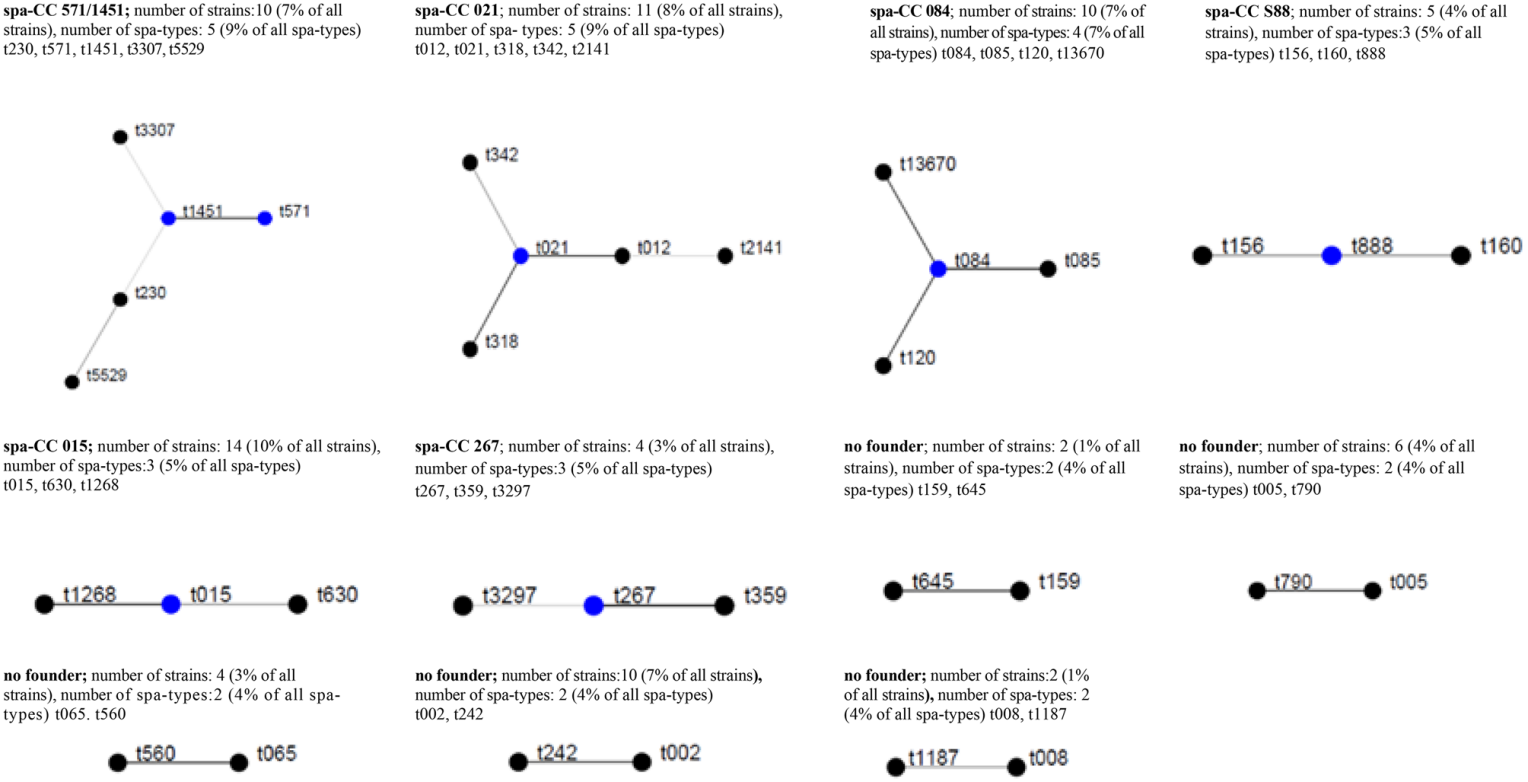
Population structure of 139 *S. aureus* oral isolates after BURP analysis with a cost of 4. Clusters of linked *spa* types correspond to *spa*-CCs. The *spa* types that were defined as founders of particular clusters are indicated in blue. % of strains based on 139 strains collection; % of spa-types based on 57 spa-types (including excluded ones).

The identified *spa* types were clustered into six *spa-*clonal complexes (*spa*-CCs) by BURP repeat analysis; *spa*-CC 571/1451, *spa*-CC 021, *spa*-CC 084, *spa*-CC 888, *spa*-CC 015, and *spa*-CC 267. Forty-six (33.1%) isolates represented 19 *spa* types and belonged to singletons, ten (7.2%) isolates were excluded from BURP cluster analysis due to presence of less than four *spa* repeats. The newly described *spa* types were distributed across various clusters and singletons (Fig. 1, Table 3).

Twelve MRSA isolates were assigned to three clonal complexes (*spa*-CC 084, *spa*-CC 888 and spa-CC 021) and five singletons, comprising of 9 *spa* types (t012, t091, t156, t189, t437, t888, t5644, t13670, t18953). Two isolates were excluded due to the low number of repeats. Particular attention should be given to two isolates of the *spa* type t437 having *lukS*-PV/*lukF*-PV genes as well as *seb* and *sek* (Table 3).

Overall, most isolates (10%) were assigned to the *spa*-CC 015 clonal complex and carried the most enterotoxin genes, *egc* (78.6%), *sec* (71.4%), *sel,* (42.8%), and *sej* (28.6%). *egc*-positive *S. aureus* isolates were not limited to a single clonal group. All isolates belonged to the *spa*-CC 021 also had superantigen genes, such as *egc (*72.7%)*, sea* (72.7%) and *tst* (27.3%). All of them showed resistance to penicillin and most to tetracycline (81.8%). Another toxigenic clonal complex, *spa*-CC571/1451 carried *egc* genes and showed inducible phenotype of erythromycin and clindamycin resistance. *spa*-CC 084 contained 7.2% of isolates, 90% of which did not have any toxin genes and were penicillin-resistant (Tables 3, 4, Fig. 1).

**Table 4:** Prevalence of toxin genes among six *spa*-clonal complexes (*spa*-CC) of oral *S. aureus* isolates.

Toxin genes	*spa*-CC 015 % (n = 14)	*spa*-CC 021 % (n = 11)	*spa*-CC 571/1451 % (n = 10)	*spa*-CC 084 % (n = 10)	*spa*-CC 888 % (n = 5)	*spa*-CC 267 % (n = 4)	p-value
*sea*	0	72.7 (8)	0	0	0	0	< 0.001
*seb*	0	0	0	0	40 (2)	0	0.001
*sec*	71.4 (10)	0	10 (1)	0	40 (2)	0	< 0.001
*seh*	0	0	10 (1)	0	0	0	0.482
*sej*	28.6 (4)	0	0	0	0	0	0.030
*sek*	0	0	0	0	40 (2)	0	0.001
*sel*	42.8 (6)	0	0	0	0	0	0.002
*egc*	78.6 (11)	72.7 (8)	50 (5)	10 (1)	0	0	< 0.001
*tst*	0	27.3 (3)	0		0	0	0.029

Most of isolates belonging to major MLST clonal complexes (CC45, 30, 5, and 22) were *egc*-positive. The differences in prevalence of *egc*-positive isolates were statistically significant (p < 0.001), as shown in Table 5.

**Table 5:** Prevalence of *egc*-positive isolates among five major MLST clonal complexes.

Clonal complex	CC45 (n = 21)	CC30 (n = 9)	CC5 (n = 9)	CC15 (n = 8)	CC22 (n = 5)	Total (100% = 52)
*egc*-positive strains	18 (85.7%)	8 (88.9%)	4 (44.4%)	1 (12.5%)	4 (80%)	35 (67.3%)
p value	< 0.001					

## Discussion

The epidemiology of *S. aureus* infections constantly changes, with novel clones emerging in various geographical regions^19,27,32^. This warrants continuous surveillance of staphylococcal isolates from different sources, especially from the oral cavity. Recent evidence suggests that the latter constitutes a significant yet underrated reservoir of *S. aureus*^10^.

*Staphylococcus aureus* isolation rates differ considerably depending on the population. In healthy adults, oral carriage rates vary from 12 to 36%, with more frequent isolation (17–48%) among students^4,33^. The carriage rate documented in our study (18.5%) was similar as reported by other authors^34–36^, and remained within a typical range for European and American adult dental patients. It was, however, lower than in dentistry students examined by Ohara-Nemoto (46.6%)^7^, and in patients with periodontitis included in another study^6^. Acrylic denture wearers also seem to be predisposed to the oral carriage^37^, with *S. aureus* isolation rates of 27–48%^2,38^.

Nearly 9% of isolates included in this study were resistant to methicillin. The prevalence of MRSA in the oral cavity of adult dental patients is known to be relatively low, 5–12%^2,5,36,39^. Smith found MRSA strains among 6% of studied patients, more often in those over 70 years^39^. Long-term MRSA carriage was also reported in 6 out of 10 complete denture wearers participating in a Finnish study, and 10.3% of Asian denture wearers^38,40^.

MRSA are typically isolated in a hospital setting; such hospital-acquired MRSA (HA-MRSA) are believed to spread primarily via human-to-human transmission^41^. However, a growing number of infections caused by non-nosocomial MRSA, referred to as community-acquired MRSA (CA-MRSA) was also observed in the last decade^42,43^. CA-MRSA genetically differ from HA-MRSA, are less resistant to non-β-lactam antibiotics and carry a smaller version of staphylococcal cassette chromosome *mec* (SCC*mec*)^44,45^. All MRSA isolates in this study harbored SCC*mec* type IV or V and were more susceptible to non-β-lactam antimicrobials, which suggested their community origin (CA-MRSA). None of the MRSA were resistant to ciprofloxacin, chloramphenicol or vancomycin. The proportions of strains resistant to gentamycin, clindamycin, and erythromycin were similar as in other types of staphylococcal infections^27,46^. The analyzed MRSA showed multidrug resistance (MDR) more often than MSSA, which is consistent with the characteristics of MRSA strains^47,48^.

*spa* typing demonstrated a broad genetic diversity of our staphylococcal isolates. We did not find any published report about the clonal variety of oral *S. aureus*. Nevertheless, the heterogeneity observed in our series corresponds well with recent European and American data about MRSA involved in other infections^49,50^. Rather than showing clustering, a typical feature of MRSA, most of our methicillin-susceptible isolates displayed extensive genetic diversity. Our MSSA corresponded to major clones widespread in Poland and other European countries^19,49,51–54^. In contrast, different predominant clonal complexes were reported in Africa and Asia, indicating geographical variation^53,55,56^.

The most toxigenic clonal complexes in our series were CC45, CC30 and CC22, which included the largest proportion of staphylococci testing positively for toxin genes in *egc* locus. The enterotoxin gene cluster (*egc*) encodes up to six enterotoxins (SEG, SEI, SEM, SEN, SEO, and SEU) being superantigens. The superantigens induce T lymphocytes and antigen-presenting cells causing massive cytokine production, with lethal effects dependent on direct toxic and cytokine effects on the cardiovascular system^57^. Recent studies showed that *egc*-clustered enterotoxins are the most prevalent virulence factors in *S. aureus* isolated nowadays^27,46,58^. However, longitudinal studies are needed to better elucidate the role of locus *egc* in oral *S. aureus* strains.

The predominant clonal complex in our material was CC45, with the most common *spa* type being *spa-*CC t015. CC45 are widely distributed among both nasal colonization and bloodstream infections strains in Europe^19,59^, and recent results suggest that the nasal isolates carry the potential to cause an invasive disease^59^. However, no reports on severe infections caused by oral CC45 strains have been published to date. Bonnet observed predominance of *spa*CC t015 among *S. aureus* associated with infective endocarditis^60^, and Deasy pointed to this *spa* type as an emerging etiological factor of bloodstream infections^19^. Other authors reported on infective endocarditis after dental extraction and treatment ^61,62^. These findings imply that peroral spread of endogenous *S. aureus* should be considered in at least some instances. Our findings suggest that *spa*-CC t015 seems to be particularly prone to the acquisition of virulence factors, including superantigens genes, *sec*, *sel, sej* and *egc*. These findings are consistent with the data from a report on bloodstream infections^63^.

The second most common clonal complex in our series was CC30. Many previous studies analyzed CC30 strains and their link with endocarditis^64^; and some authors demonstrated their role in the development of an invasive disease^19,20^. Our *spa*-CC 021 belonging to CC30 clone characterized high proportion of *egc*-, *sea*-and *tst*-positive strains, especially CC30-t012, which is consistent with the results of previous studies^28,52,65^. Also, according to an American report, CC30 strains from patients with infective endocarditis were significantly more likely to contain these enterotoxin genes and had the potential to cause hematogenous complications^64^.The high resistance rates to penicillin and tetracycline was observed among our CC30 strains. To this date, resistance to tetracycline was not reported in *S. aureus* from the oral cavity but in the strains from other human or animal sources^66–70^. However, tetracycline is also used in the treatment of some oral infections in humans, especially in patients with periodontitis^71^. Thus, our observation on the potential emergence of tetracycline-resistant oral strains warrants further investigation.

To the best of our knowledge, present study was the first to demonstrate the prevalence of *spa* type t437 SCC*mec*-V-*pvl*-positive *S. aureus* strains in dental patients. Panton-Valentine leukocidin (PVL) genes are considered a stable genetic marker for CA-MRSA strains carrying SCCmec type IV or V^72,73^. More than half (66.6%) of strains identified in this study were assigned to SCC*mec* IV, whereas the strains represented *spa* t437 harbored SCC*mec* V. Similar strains were isolated in Germany and Taiwan, and according to a recent Polish report, t437 SCC*mec*-IV-*pvl*-positive strains predominated in specimens from diabetic patients^74–76^. The PVL -positive strains were associated with purulent skin infections, necrotizing pneumonia, pyomyositis and other *S. aureus* infections^74,77–79^. PV leukocidin is considered a potent inducer of inflammation and cytotoxicity but its role in oral infections is still little unknown^80,81^.

Our study has several limitations. First, the proportion of MRSA strains was small in comparison with number of MSSA strains. It should be taken account in the interpretation of the results. Second, we did not analyze clinical data of the swabbed patients, such as type of oral infection and its manifestations. However, even considering these drawbacks, the results of our study add to current knowledge about oral *S. aureus* strains.

In conclusion, this study demonstrated that both methicillin-susceptible and methicillin-resistant *S. aureus* strains major European clones could be isolated from the oral cavity of dental patients, with the emergence of PVL-positive CA-MRSA strains. The oral cavity should be considered as a possible source of toxigenic *egc-*positive *S. aureus* strains, in terms of potential risk of cross-infection and dissemination to other body sites.

## Materials and methods

### Isolation and identification of *S. aureus*

The study included oral *S. aureus* isolated from all 2327 oral microbiological samples analysed consecutively at the Laboratory of Department of Oral Microbiology of the Medical University of Gdansk during routine clinical laboratory procedures, over a period of three years. The samples were obtained with sterile cotton swabs from the oral mucosa, the dorsal surface of the tongue, denture surface and angular cheilitis lesions. The analysed *S. aureus* were not specifically isolated for this research, they were part of the diagnostic laboratory procedure and no humans were involved in the experiments.

All samples were plated onto Columbia blood agar (GrasoBiotech, Starogard Gd., Poland) and mannitol salt agar (bioMérieux, Marcy l'Etoile, France) and were incubated 18–24 h at 37 °C. Suspected staphylococcal colonies were identified by standard methods, on the basis of colony characteristics, pigment production, Gram-staining, haemolysis and Pastorex StaphPlus latex agglutination kit (Bio-Rad, Marnes la Coquette, France). Further, all isolates eventually identified as *S. aureus* based on PCR amplification of species-specific thermostable nuclease gene (*nuc*)^82^.

After final identification, the isolates were stored at − 80 °C in Trypticase Soy Broth (Becton Dickinson, Franklin Lakes, NJ, USA) supplemented with 20% glycerol.

### Antimicrobial susceptibility testing

The antimicrobial susceptibility was determined on Mueller–Hinton agar plates (Becton Dickinson, Franklin Lakes, NJ, USA) by the disk diffusion method and interpreted according to the EUCAST^83^. The following antimicrobial agents were tested: oxacillin, cefoxitin, gentamicin, erythromycin, clindamycin, tetracycline, chloramphenicol, ciprofloxacin, amoxicillin/clavulanic acid, trimethoprim/sulfamethoxazole (Bio-Rad, Marnes la Coquette, France) and penicillin G (Oxoid, Basingstoke, England). The inducible resistance to macrolide-lincosamide-streptogramin B (MLS_B_) was detected by disk diffusion method with use the clindamycin (2 μg) and erythromycin (15 μg) disks positioned 15–26 mm apart^83^. Resistance to gentamicin was verified by using E-tests (bioMérieux, Marcy l'Etoile, France), an isolate with MIC value > 1 µg/ml was considered as a resistant^83^. Vancomycin susceptibility was determined with E-test strips (bioMerieux, Marcy-l’Etoile, France), in line with the manufacturer’s instruction. Multidrug resistance (MDR) was defined as a resistance to three or more classes of antimicrobials. Resistance to methicillin was first identified using cefoxitin (30 µg) and oxacillin (1 µg) disks, and then confirmed by the detection of PBP2a protein (Latex Agglutination Test Kit, Oxoid, Basingstoke, England), and verified by the detection of the *mec*A gene according to Khairalla et al.^25^ and *mec*C according to Stegger et al.^84^. *S. aureus* ATCC25923 (methicillin-susceptible) and *S. aureus* ATCC43300 (MDR) were used as the reference strains.

### Molecular characterization

#### Isolation of staphylococcal DNA

Genomic Micro AX Staphylococcus Gravity kit (A&A Biotechnology, Gdynia, Poland) was used to isolate genomic DNA from bacteria by gravity according to the manufacturer's instructions.

### Detection of toxin genes

Genes of the enterotoxins (*sea, seb, sec, sed, see*), toxic shock syndrome toxin-1 (*tst*), and exfoliative toxins (*eta, etb*) were detected as described by Becker et al.^85^, for the other enterotoxins genes (*seg, seh, sei, sej, sek, sel, sem, sen, seo, seu*) according to Bania et al.^86^. Detection of Panton-Valentine leukocidin genes (*lukS*-PV/*lukF*-PV) was performed as described by Lina et al.^87^.

### SCC*mec* typing

Typing of the five (I–V) major staphylococcal chromosomal cassette *mec* (SCC*mec*) in MRSA strains was determined by PCR as described by Oliveira et al.^88^ and by Milheiriço et al.^89^. The SCC*mec* type was determined on the basis of the band pattern profiles obtained.

### *spa* typing

The *spa* typing, based on amplification of the variable X region of protein A gene, was performed as described previously^90^. The spa types were assigned using the Ridom StaphType software version 2.2.1 (https://www.ridom.de/ Ridom GmbH, Wurzburg, Germany) and the Ridom SpaServer database (https://spaserver.ridom.de/). The predicted MLST were assigned based on Ridom SpaServer. The based upon repeat pattern (BURP) algorithm was used to calculate spa clonal complexes (*spa*-CCs) with following parameters: (I) exclude *spa* types shorter than 5 repeats; (II) cost less or equal to 4; (III) cluster composed of 2 or more related *spa* types was regarded as CC; (IV) a *spa* type that was not grouped into a CC was considered as singleton.

### Statistical analysis

All calculations were performed with Statistica 10 package (StatSoft, Tulsa, OK, USA). The significance of between-group differences in the percentages of positive isolates was verified with Pearson chi-squared test or Fisher exact test. The threshold of statistical significance was set at p ≤ 0.05, with Bonferroni correction applied whenever multiple comparisons had to be carried out.
